# Oncology Specialists' Perceptions and Insights Into Dihydropyrimidine Dehydrogenase Testing in Palestine

**DOI:** 10.1002/cnr2.70251

**Published:** 2025-06-24

**Authors:** Mohammad Dweib, Hussein Hallak, Hani Hour, Asmahan Alzeer, Raya Fatafta, Leen Albaw

**Affiliations:** ^1^ College of Public Health Al‐Quds University Jerusalem Palestine; ^2^ College of Pharmacy and Medical Sciences Hebron University Hebron Palestine; ^3^ College of Pharmacy Al‐Quds University Jerusalem Palestine; ^4^ Palestinian Ministry of Health Ramalla Palestine

**Keywords:** cancer, capecitabine, fluoropyrimidines, genetic testing, pharmacogenomics

## Abstract

**Background:**

This study evaluated awareness, prevalence, and utilization of dihydropyrimidine dehydrogenase (DPD) testing and pharmacogenomics among oncologists, residents, and clinical pharmacists working in Palestinian hospitals.

**Aim:**

This study aimed to assess the knowledge and opinions of HCPsspecializing in oncology in Palestine regarding screening for DPYD testing prior to prescribing FP.

**Methods:**

A cross‐sectional survey was distributed to 106 HCPs across various hospitals in Palestine.

**Results:**

A notable deficiency in training and implementation of pharmacogenomics was observed, with over 70% of participants lacking formal training in the field. Although there is high awareness of DPD deficiency and its impact, fewer than 50% of participants screen for DPD deficiency prior to prescribing fluoropyrimidines (FP). Standardization and promotion of DPD testing are low, and guidelines for prescribing FP are lacking, leading to variations in clinical practice.

**Conclusion:**

These findings highlight the need for enhanced training, standardized protocols, and increased awareness to improve patient safety and outcomes in cancer treatment in Palestine.

## 1 Introduction

Capecitabine and 5‐fluorouracil are chemotherapeutic agents belonging to the FP class. These agents are commonly used for the treatment of several cancers, such as colorectal and breast cancer [1, 2].

It has been shown that a significant proportion of patients receiving these agents, ranging from 10% to 40% of people, experience severe and potentially life‐threatening toxicity in the initial stages of FP treatment [3, 4]. Toxicity can lead to the disruption or cessation of treatment. In severe cases, it may require emergency department visits or hospital admissions [5, 6, 7].

Reduced activity of the DPD enzyme, which metabolizes around 80%–90% of the administered 5‐FU to inactive 5,6‐dihydro‐5‐fluorouracil and is the rate‐controlling enzyme for the inactivation of 5‐FU, primarily encoded by the DPD gene (DPYD), is considered among the genetic factors implicated in the development of FP intolerance, attracting considerable attention in scientific research, especially clinical research focusing on toxicity prediction [8]. DPD deficiency leads to toxicity by impairing the metabolism of 5‐FU, preventing its breakdown into the inactive metabolite 5,6‐dihydro‐5‐FU. Consequently, 5‐FU accumulates in the body, causing excessive levels of its active metabolites (FdUMP, FdUDP, and FdUTP). These metabolites interfere with DNA replication and RNA synthesis, leading to cytotoxic effects in rapidly dividing cells. This disruption causes severe toxicities, including myelosuppression, neurotoxicity, and gastrointestinal damage, which can be life‐threatening in individuals who have partial or complete DPD deficiency [9]. The categorization of individuals according to their DPYD genotype holds promise in mitigating the occurrence of unfavorable outcomes [10]. This led to the recommendation of DPYD testing prior to treatment with these medications in most parts of Europe and Canada [11].

In addition to genetic factors, other factors, such as age, comorbidities, concurrent medications, and treatment cycles, have also been studied for their role in FP‐induced toxicity. Notably, variables such as patient age, the presence of metastasis, the presence of comorbidities, using other medications, and the number of treatment cycles have been explored and have revealed associations with treatment‐related toxicity [12].

In many high‐income countries, such as those in Europe and Canada, DPD deficiency testing is increasingly integrated into routine clinical practice before administering 5‐FU‐based chemotherapy, as recommended by the European Society for Medical Oncology (ESMO) guidelines. These guidelines emphasize the importance of screening patients for *DPYD* gene mutations to prevent severe toxicity, particularly in colorectal cancer treatment [13, 14].

Recent studies have further highlighted the significance of integrating pharmacogenomic testing into routine oncology practice, particularly in screening for DPYD variants. Despite the well‐documented risks associated with DPYD deficiency, routine pretreatment testing remains inconsistent across different healthcare systems due to logistical, financial, and regulatory challenges [15]. The implementation of systematic DPYD screening has been shown to enhance patient safety while being cost‐effective by reducing severe adverse drug reactions and hospitalizations. Large‐scale pharmacogenomics analyses have further demonstrated that preemptive DPYD genotyping significantly decreases treatment‐related complications and aligns with the broader shift toward personalized medicine [16].

Efforts to standardize DPYD testing across different regions have gained momentum. In Europe, pharmacogenomic guidelines for fluoropyrimidine chemotherapy have led to increased accessibility and reduced toxicity rates [17]. Similarly, the Pharmacogenomics Global Research Network has been working to promote the adoption of DPYD testing globally, aiming to ensure equitable access to pharmacogenomic interventions [18]. However, differences in testing rates persist, underscoring the need for stronger policy frameworks and clinician education to maximize the clinical benefits of DPYD screening [19].

Expanding the role of healthcare professionals (HCPs), particularly pharmacists, in implementing pharmacogenomic testing may further support the routine integration of DPYD testing in oncology settings. Pharmacists have been recognized as key stakeholders in advancing pharmacogenomic initiatives due to their expertise in medication management and patient counseling [20]. Moreover, targeted educational programs for oncologists and pharmacists have shown promising results in increasing awareness and acceptance of DPYD testing as a standard component of fluoropyrimidine therapy [21]. A multidisciplinary approach—incorporating standardized guidelines, institutional support, and education—is crucial for successfully embedding DPYD screening into routine clinical practice.

Recent advancements in fluoropyrimidine drug development have introduced CF10, a second‐generation fluoropyrimidine polymer designed to enhance efficacy while reducing systemic toxicity. Preclinical studies have shown that CF10 is well‐tolerated and remains effective even in models simulating DPD inhibition, suggesting its potential as a safer alternative for patients with DPD deficiency. This emerging therapy may help mitigate the risks associated with fluoropyrimidine chemotherapy and could serve as an alternative approach in resource‐limited settings where routine DPYD testing is not widely available [22, 23].

Despite the importance of DPYD genotyping and the correlation of other factors with FP toxicity in other populations [24, 25, 26], there is a significant gap in our understanding concerning its applicability and significance in the context of Palestinian cancer patients. To date, no studies have conducted a comprehensive examination of the prevalence of DPYD genetic variations or other clinical factors and their correlation with FP‐related toxicities within this specific population.

This study aimed to assess the knowledge and opinions of HCPsspecializing in oncology in Palestine regarding screening for DPYD testing prior to prescribing FP. The specific objectives of this study were:

(1) to evaluate the awareness of HCPs (oncologists, residents, and clinical pharmacists) in Palestine with pharmacogenetic screening in general and DPYD screening tests in particular before administering FP to cancer patients;

(2) to evaluate if demographic variables such as age, hospital department, duration of work experience, and current designation affect awareness;

(3) to assess the current prevalence, utilization, and barriers to implementing *DPYD* testing and pharmacogenomics in Palestine.

## 2 Materials and Methods

This cross‐sectional study utilized a survey distributed to oncologists, oncology residents, and clinical pharmacists across various hospitals in Palestine between February and May 2024. The questionnaire was adopted from a previous study conducted in Saudi Arabia [21] and was slightly modified to suit the Palestinian context. These modifications included, for example, the type of healthcare provider in which the respondents worked (Ministry of Health, private sector, UNRWA, etc.)The survey consisted of 26 items covering participant demographics and the awareness of participants regarding DPYD screening. Various types of questions were included (multiple choice questions, Likert scale, etc.) The content validity of the questionnaire items was ensured through review by experts (three oncologists and three pharmacists) who assessed the appropriateness of the content, making necessary modifications to ensure comprehensive and accurate assessment of attitudes.

The survey was pilot‐tested by four professionals (two oncologists and two pharmacists). No major improvements were suggested, so no modifications were performed. Their responses were not included in the analysis.

The questionnaire was distributed electronically to all available health care professionals who work in four hospitals (two governmental, two nongovernmental) that have an oncology unit.

### 2.1 Inclusion Criteria

All oncologists, oncology residents, and clinical pharmacists in the four cancer centers in Palestine. The number of professionals who met the criteria in the hospitals was 171.

### 2.2 Exclusion Criteria

Retail pharmacists, outpatient pharmacists, and nononcology doctors. Retail pharmacists were excluded because no anticancer medications are dispensed through them.

### 2.3 Sample Size

Prior studies have shown that the level of knowledge among HCPs on pharmacogenomics testing varied between 31% and 62% [27, 28, 29, 30]. For our study conducted in Palestine, we made the assumption that the level of awareness of DPD testing is roughly 50%. To achieve the required precision level of 5% and a confidence interval of 95%, and taking into account the information provided by the Palestinian health authorities that there are around 150 registered practitioners in oncology, we needed to recruit 100 HCPs in order to meet the aim of our study. Incomplete responses were not included in the analysis.

## 3 Results

The questionnaire was completed by a total of 106 HCPs, consisting of 57.5% specialist physicians, 27.4% clinical pharmacists, and 15.1% resident physicians. This distribution is similar to composition of the study population. The majority of respondents, over 60%, received their most recent degree from a Palestinian institution, while around 40% graduated from foreign institutions. The location of the last degree may influence respondents' practices due to variations in pharmacogenomics training and exposure to DPYD testing. Foreign institutions may emphasize pharmacogenetic applications more than Palestinian institutions, leading to differences in implementation.

More than 70% of the participants were 45 years old or younger.

Regarding professional experience, a majority of the participants possessed more than 8 years of expertise in working with cancer patients as seen in Table 1. Fifty percent of the participants were associated with private hospitals, while the remaining 50% were employed at governmental institutions.

**TABLE 1:** Sociodemographic characteristics of the participants.

Characteristics		Number	Percentage
Designation	Clinical pharmacist	29	27.4
	Physician	61	57.5
	Resident	16	15.1
Last degree location	Inside Palestine	63	59.4
	Outside Palestine	43	40.6
Age group	23–35 years	41	38.7
	36–45 years	37	34.9
	46–59 years	22	20.8
	More than 60 years	6	5.7
Years of experience	2–4	25	23.6
	5–7	22	20.8
	8–10	14	13.2
	11 or more	45	42.5
Hospital type	Private	54	50.9
	Governmental	52	49.1

Demographic data for the participants is presented in Table 1.

A striking finding was that more than 70% of participants stated that they had never undergone formal training in pharmacogenomics during their career. However, a substantial majority of 75.5% stated that they usually prescribeFPs. Furthermore, around 80% of participants indicated knowledge of the mutation linked to DPD deficiency and its consequences for FP toxicity. Yet, fewer than 50% of participants indicated that they consistently or occasionally screen for DPD deficiency prior to prescriptionFPs. This discrepancy between awareness of DPYD mutations and the limited use of screening has significant implications for patient outcomes. Failure to screen for DPD deficiency may lead to severe, potentially life‐threatening toxicities in patients prescribed FPs, as individuals with DPYD mutations are unable to metabolize these drugs effectively. Implementing routine screening could enhance treatment efficacy by identifying at‐risk patients, enabling dose adjustments or alternative therapies, and reducing the risk of adverse events.

In addition, only 47% of participants reported that their institution had specific protocols for prescribing FPs. Notably, 60% of individuals experienced significant toxicity associated with FPs, a rate considerably higher than the global average of 10%–30%. This difference may be influenced by several factors, including variations in dosing strategies, differences in patient monitoring, and inconsistent adherence to international treatment guidelines. Furthermore, only 33% of participants had observed the influence of DPD testing in clinical practice, highlighting gaps in implementation. These challenges may be driven by the lack of infrastructure for pharmacogenomic testing, limited awareness of standardized protocols, and financial constraints that hinder the widespread adoption of DPD testing. Addressing these barriers could improve the safe and effective use of fluoropyrimidines in clinical practice. With regards to the standardization and promotion of DPD testing, a mere 5.7% and 6.6% of participants, respectively, reported the existence of standardized DPD testing and the Palestinian Ministry of Health's promotion of DPD testing before prescribing FPs. Only 1.9% of the participants concurred that an adequate amount of research is being conducted in Palestine with regards to DPD insufficiency.

The main sources used to obtain information on specific molecular testing were published practice guidelines (37.7%) and cancer‐genomics specific guidelines (13.2%).

The most common types of cancer for which FPs were prescribed were colorectal cancer (29%) and breast cancer (27%).

Details of the awareness and practices of the participants are shown in Table 2.

**TABLE 2:** Practices, awareness, and attitudes of participants toward DPD testing.

		Count	Percentage
Do you participate in tumor genomic board, tumor molecular board in your current institution?	No	67	63.2
	Yes	39	36.8
What do you mostly follow as a reference when ordering a specific molecular test?	Cancer‐genomics specific guidelines	24	22.64
	Institutional guidelines	17	16.04
	Personal experience	20	18.87
	Published practice guidelines	45	42.45
Have you received any formal training or education in pharmacogenomics?	No	77	72.6
	Yes	29	27.4
If yes, where did you receive your education or training in pharmacogenomics?	Continuous medical education (CME)	21	19.81
	Experience through practice	18	16.98
	Fellowship program	13	12.26
	Hospital seminars	16	15.09
	Online courses	16	15.09
	Research	7	6.60
	University education	15	14.15
Do you usually prescribe FP (5‐fluorouracil [5‐FU] and Capecitabine) for patients with cancer?	No	26	24.5
	Yes	80	75.5
If yes; which type of cancer would you prescribe it for?	Colorectal cancer	21	19.81
	Breast cancer	19	17.92
	Colorectal cancer	14	13.21
	Gastric cancer	13	12.26
	Pancreatic cancer	7	6.60
	Head and neck cancer	8	7.55
	Biliary tree malignancy	9	8.49
	I am not prescriber	11	10.38
	Nasopharyngeal carcinoma	4	3.77
Are you aware of a mutation that causes DPD deficiency?	No	21	19.8
	Yes	85	80.2
If yes; are you aware of its complications; e.g., PD toxicity of FP (5‐fluorouracil [5‐FU] and Capecitabine)?	No	22	20.8
	Yes	84	79.2
How often do you screen for DPD deficiency prior to prescribing FP?	Always	2	1.9
	Never	56	52.8
	Sometimes	48	45.3
Are there guidelines for prescribing FP in your hospital?	No	27	25.5
	Not sure	29	27.4
	Yes	50	47.2
Is there enough research being done in Palestine on screening for DPD prior to prescribing FP?	No	62	58.5
	Not sure	42	39.6
	Yes	2	1.9
Have you seen patients with severe toxicity related to use of FP?	No	41	38.7
	Yes	65	61.3
Have you seen the impact of DPD testing in your clinical practice?	No	71	67.0
	Yes	35	33.0
Is there any standardization of DPD Deficiency practice done by MOH?	No	51	48.1
	Not sure	49	46.2
	Yes	6	5.7
Does the MOH promote the practice of DPD deficiency test prior to FP group of medications?	No	51	48.1
	Not sure	48	45.3
	Yes	7	6.6

A high percentage of participants displayed knowledge regarding the mutation accountable for DPD deficiency. Specifically, 72% of clinical pharmacists, 80% of physicians, and 93% of resident physicians expressed familiarity with this genetic variation (*p* > 0.05).

The awareness of the mutation linked to DPD deficiency differed among respondents with different degrees of professional experience. Specifically, 84% of those with 2–4 years of experience, 90% with 5–7 years, 64% with 8–10 years, and 77% with 11 or more years indicated familiarity with this genetic variant (*p* > 0.05).

An inequality in knowledge regarding the mutation accountable for DPD was noted between HCPs employed in private hospitals and those in governmental hospitals, with 87% of individuals in private hospital settings and 73% in governmental hospitals expressing familiarity with this genetic variant (*p* > 0.05).

Some participants reported the existence of guidelines in their individual institutions for prescribing FPs. Specifically, 37% of clinical pharmacists, 50% of physicians, and 50% of residents confirmed the presence of these guidelines (*p* > 0.05).

The presence of guidelines for prescribing FPs in hospitals varied based on years of professional experience. Among individuals with 2–4 years of experience, 52% indicated the existence of guidelines. This percentage was 45% for those with 5–7 years, 57% for those with 8–10 years, and 42% for those with 11 or more years (*p* > 0.05).

There was a noticeable difference in the reported prevalence of guidelines for prescribing FPs between HCPs in private hospitals and those in governmental hospitals. In private hospital settings, 57% of individuals acknowledged the presence of these guidelines, while in governmental hospitals, only 36% did so (*p* > 0.05).

A small proportion of participants stated that they had witnessed the effects of DPD testing in their clinical work. Specifically, 17% of clinical pharmacists, 39% of physicians, and 37% of residents reported having this experience (*p* > 0.05).

Regarding professional experience, the extent to which the effects of DPD testing were observed in clinical practice differed among individuals. Specifically, 28% of those with 2–4 years of experience, 40% with 5–7 years, 28% with 8–10 years, and 33% with 11 or more years reported witnessing such influence (*p* > 0.05).

A statistically significant difference was found between HCPs working in private hospitals and those working in governmental institutions in terms of their perception of the influence of DPD testing in clinical practice, with a *p* = 0.001. More precisely, 48% of persons in private hospital settings and 17% in governmental institutions reported experiencing this effect (Table 3). Additionally, the data from Table 3 reveal that awareness of the DPD mutation was highest among residents (93.8%) and private hospital practitioners (87.0%). However, the presence of hospital‐specific guidelines was reported more frequently in private hospitals (57.4%) than in governmental settings (36.5%), further reflecting institutional differences in support for fFP‐related testing. These findings highlight the need for standardized policies across all hospital types to improve the adoption of DPD testing in clinical practice.

**TABLE 3:** Knowledge of and observation of DPD testing in practice, determined by hospital‐ and demographic‐related factors.

			Are you aware of a mutation that causes DPD deficiency	Are there guidelines for prescribing Fluoropyrimidine in your hospital?	Have you seen the impact of DPD testing in your clinical practice?
Designation	Clinical Pharmacist	29	21 (72.4%)	11 (37.9%)	5 (17.2%)
	Physician	61	49 (80.3%)	31 (50.8%)	24 (39.3%)
	Resident	16	15 (93.8%)	8 (50.0%)	6 (37.5%)
Years of experience	2–4	25	21 (84.0%)	13 (52.0%)	7 (28.0%)
	5–7	22	20 (90.9%)	10 (45.5%)	9 (40.9%)
	8–10	14	9 (64.3%)	8 (57.1%)	4 (28.6%)
	11+	45	35 (77.8%)	19 (42.2%)	15 (33.3%)
Hospital type	Private	54	47 (87.0%)	31 (57.4%)	26 (48.1%)
	Governmental	52	38 (73.1%)	19 (36.5%)	9 (17.3%)

The institution where HCPs obtained their most recent degree did not have a significant impact on their knowledge of DPD mutations, awareness of institutional guidelines for DPD testing, or experience with its effects in clinical practice, as indicated by a *p* > 0.05. Details of the awareness and observation of DPD testing in practice, with regard to hospital‐related and demographic variables, are shown in Table 3.

## 4 Discussion

The result emphasizes a notable deficiency in training regarding pharmacogenomics and implementation of pharmacogenomic testing among HCPs. Although most participants indicate that they have not received formal training in pharmacogenomics, no differences were observed between roles, such as residents, clinical pharmacists, and physicians. Despite this lack of training across all groups, a significant percentage still administer FPs, underscoring the need for comprehensive educational initiatives to improve safety and efficacy in clinical practice. This difference indicates that numerous HCPs may lack comprehensive knowledge regarding the complex nature of pharmacogenomics and its implications for drug therapy. This gap highlights the urgent need for targeted educational initiatives and training programs to bridge the knowledge deficit amongHCPs. Incorporating pharmacogenomics into medical and pharmacy curricula, as well as offering continuing education opportunities, could enhance practitioners' understanding and improve the safe and effective use of FPs. This is particularly critical given the potential for severe adverse drug reactions in patients with DPYD gene variants.

It is encouraging to notice that almost 80% of HCPs have a high level of awareness about the mutation linked to DPD deficiency and its impact on fluoropyrimidine toxicity. This suggests that healthcare practitioners are generally well‐informed about this important genetic component. Nevertheless, it is worrisome that fewer than 50% of the participants stated that they screen for DPD deficiency prior to prescribing FPs. This gap between awareness and practice may be attributed to financial and logistical barriers, such as the high cost of DPD testing, limited availability of testing facilities, and a lack of institutional protocols, which hinder the integration of pharmacogenetic testing into routine clinical practice. The lack of implementation in this domain may result in a higher likelihood of severe toxicity in patients with DPD deficiency who receive FPs.

The indication that fewer than 50% of the participants reported their institution having explicit standards for administering FPs implies a deficiency in uniformity within this domain. This lack of standardized protocols can lead to inconsistent practices, increasing the risk of adverse drug reactions and compromising patient safety. Without clear guidelines, HCPs may fail to screen for DPD deficiency, resulting in suboptimal dosing or inappropriate treatment that could reduce treatment efficacy and expose patients to potentially severe toxicity. The absence of clear recommendations may lead to variations in prescribing practices and potentially result in inferior outcomes for patients. For example, some practitioners may omit screening for DPD deficiency altogether, leading to standard dosing that could cause severe toxicity in patients with the mutation, while others might delay treatment adjustments due to uncertainty or lack of access to testing. Such inconsistencies can compromise both patient safety and the overall efficacy of FP‐based therapies.

The significant incidence of serious toxicity associated with FPs (about 60%) is alarming and emphasizes the necessity for enhanced management measures. This rate appears to be higher than the global average, where severe toxicity typically ranges between 10% and 30% in patients receiving FPs. Such a stark contrast highlights potential gaps in screening practices, dosing adjustments, or patient monitoring in the local context, underscoring the urgent need for standardized protocols and broader implementation of DPD deficiency testing. One method to reduce this risk is through DPYD testing, which can identify patients who have a higher chance of experiencing toxicity as a result of DPD deficiency. Nevertheless, the very low percentage of observations regarding the influence of DPD testing in clinical practice (merely 33%) implies that this testing may not be extensively adopted or acknowledged as being beneficial among HCPs. To improve adoption, strategies such as integrating DPD testing into national treatment guidelines, subsidizing the cost of testing to reduce financial barriers, and conducting educational campaigns to raise awareness about its clinical value could be implemented. Additionally, policies mandating the inclusion of pharmacogenomics in medical and pharmacy curricula and incentivizing healthcare institutions to adopt routine screening protocols may further promote the use of DPD testing.

The low rates of standardization of DPD testing (5.7%) and promotion by the Ministry of Health (6.6%) underscore the necessity for heightened awareness and implementation of this crucial component of personalized medical care. Implementing standardized protocols and actively promoting DPYD testing has the potential to enhance uniformity in healthcare institutions and ultimately enhance patient outcomes.

This indicates a lower‐than‐anticipated adoption of a screening test that is already regarded as a standard procedure in numerous other countries like France and the Netherlands [9, 31]. Consequently, our findings emphasize the significance of promoting DPD screening prior to the administration of fluoropyrimidines.

The results uncover a significant pattern in the knowledge of the mutation associated with DPD insufficiency across HCPs. Resident physicians demonstrated the greatest level of familiarity, with clinical pharmacists and physicians following closely behind. Curiously, there was a variation in awareness depending on professional experience, with individuals in the intermediate range (5–7 years) demonstrating the highest level of familiarity. The variance seen could be ascribed to the evolution of training methods or the influence of emerging research at this particular phase of their professional lives. Moreover, the discrepancy in understanding between private and governmental hospital settings implies possible variations in instructional tools or focus on pharmacogenomics in these settings.

The data reveals a notable difference in the existence of guidelines for prescribing FPs among various categories of HCPs and hospital environments. Guideline availability varied across clinical pharmacists, physicians, and residents, with physicians and residents being more likely to have guidelines compared to clinical pharmacists. The difference can be attributed to the inherent characteristics of their positions and duties within the healthcare system, since physicians and residents typically exhibit greater direct engagement in making prescription decisions in comparison to clinical pharmacists. Additionally, differences in funding, institutional priorities, and resource allocation between hospital environments may further contribute to this variation. Private hospitals, for example, may have more resources to develop and implement comprehensive guidelines, whereas governmental institutions may face budget constraints and competing healthcare priorities.

Curiously, the correlation between the presence of guidelines and years of professional experience was not consistently observed, contrary to what one might anticipate. This implies that factors beyond experience, like institutional agendas, resources, or the importance given to standardizing prescribing practices, might influence the creation or execution of these guidelines.

The noticeable difference in the availability of guidelines between private and governmental hospital settings highlights the impact of institutional factors on the existence of prescribing guidelines. Private hospitals are more likely to have established guidelines compared to public hospitals due to their greater resources and emphasis on quality improvement programs.

Consequently, these findings emphasize the importance of having clear and easily available guidelines for prescribing fluoropyrimidines in all healthcare environments, regardless of the practitioner's level of expertise or type of facility. These suggestions can assist in guaranteeing secure and efficient prescription practices, eventually enhancing patient results.

The analysis indicates a difference in how various groups of HCPs perceive the effects of DPD testing in clinical practice. Clinical pharmacists, physicians, and residents had varying degrees of expertise in observing the impact of DPD testing. Among them, physicians had the highest percentage of respondents who reported having such experience. The variation in results can be linked to differences in roles and duties, since physicians are more inclined to have direct participation in patient care decisions where DPD testing has an effect on treatment options. To address this variation, targeted training programs on pharmacogenomic applications and the clinical importance of DPD testing should be developed for all healthcare roles. Additionally, implementing policies that promote interdisciplinary collaboration could help ensure that all practitioners are equally informed and involved in decisions related to DPD testing.

Interestingly, the correlation between the observation of the impact of DPD testing and years of work experience was not consistently positive. This implies that variables other than experience, such as exposure to training or access to resources, can affect the probability of detecting the effects of DPD testing in clinical practice. To address this issue, targeted training programs focused on pharmacogenomics and the clinical applications of DPD testing should be implemented across all experience levels. Additionally, ensuring equitable access to testing facilities and resources within healthcare institutions can help bridge this gap and improve the integration of DPD testing into routine practice.

The observed statistically significant difference between HCPs in private and public institutions in terms of their observation of the impact of DPD testing underscores the role of institutional determinants. Private hospitals may possess stronger resources and prioritize individualized medicine, resulting in a greater probability of witnessing the effects of DPD testing in comparison to government hospitals.

The results of this study closely correspond to the findings of studies carried out in nearby nations. A study carried out in Saudi Arabia also found a lack of proper genetic testing before administering FPs, as well as a lack of awareness about established recommendations for genetic testing [21].

Research conducted in the United States revealed that although the majority of oncologists recognize the heightened risk of toxicity in patients with DPD deficiency and are open to adjusting the dose of FP appropriately, only a small percentage of them believe in the effectiveness of DPYD testing. Just 20% of oncologists have ever requested the test, and only 3% have ordered it for at least 10% of their patients receiving FP treatment. The limited occurrence of DPD insufficiency and the absence of guideline recommendations are significant obstacles to broader acceptance and implementation [32].

The comparison to similar studies in Saudi Arabia and the United States provides valuable context for understanding global challenges in implementing DPD testing. Cultural and economic factors may contribute to these similarities and differences. For instance, in Saudi Arabia, a lack of widespread genetic literacy and reliance on traditional practices may hinder awareness and adoption of genetic testing. In the United States, despite higher access to advanced technologies, skepticism about the cost‐effectiveness of testing and varying guideline recommendations may explain the limited use of DPYD testing. These findings underscore the need for tailored strategies, such as improving awareness, aligning guidelines, and addressing cultural and economic barriers, to enhance the adoption of DPD testing in diverse healthcare systems.

The cost of DPD testing includes not only the direct expenses of genetic analysis but also the infrastructural investments required for laboratory capacity, equipment, and trained personnel. In Palestine, the lack of established pharmacogenomics programs and limited access to specialized genetic testing facilities further complicate implementation. Additionally, regulatory barriers, such as the absence of national guidelines or policies mandating pretreatment DPD screening, contribute to inconsistent adoption across healthcare institutions. Overcoming these challenges will require coordinated efforts to integrate pharmacogenomics into clinical practice, improve healthcare infrastructure, and establish clear regulatory frameworks to support the safe and cost‐effective use of FP‐based therapies.

A study evaluating the application of DPD deficiency testing in Europe, both before and after the recommendations made by the European Medicines Agency (EMA), revealed a rise in the utilization of both genotyping and phenotyping techniques following the guidelines. The implementation of municipal norms has also seen an increase. In 2021, the reimbursement coverage for both tests experienced an improvement, with just a limited number of nations not providing coverage. Although significant obstacles persisted, a smaller number of experts mentioned concerns around reimbursement and recognition after the suggestions were made. In general, 25% of respondents indicated that they encountered no obstacles in implementing the new testing procedure after the recommendations were made [33]. These European practices could serve as a model for Palestine by demonstrating the value of clear national guidelines, improved reimbursement policies, and increased advocacy for testing. Adapting these strategies in Palestine would require collaboration between healthcare policymakers, institutions, and the Ministry of Health to establish unified protocols and allocate resources to make DPD testing more accessible and feasible.

The development of second‐generation FPs alternatives like CF10, which exhibit lower systemic toxicity and greater antitumor activity, aligns with the goals of reducing the burden of adverse events associated with current treatment regimens. These advancements may also reduce reliance on DPD testing, especially in resource‐limited settings where implementation barriers persist. However, integration of such therapies into clinical practice would still require robust training, guideline updates, and resource allocation to ensure equitable access and effective use [22].

Emerging research into innovative FP formulations offers promising solutions to mitigate toxicity while enhancing efficacy. For instance, Okechukwu et al. (2024) demonstrated that CF10, a second‐generation FP [22].

To address the identified gaps, it is imperative to implement actionable measures aimed at enhancing the safe and effective use of FPs. National training programs on pharmacogenomics should be established to equip HCPs with the knowledge necessary to integrate genetic testing, such as DPD deficiency screening, into routine practice. Additionally, integrating DPD testing protocols into existing healthcare systems and making them a mandatory component of treatment guidelines could standardize practices and reduce toxicity risks. These initiatives, coupled with efforts to subsidize the cost of testing and improve accessibility, would significantly enhance patient safety and optimize treatment outcomes.

Policy changes proposed to the Ministry of Health could include mandating DPD testing as a standard pretreatment requirement for FPs, subsidizing testing costs to improve accessibility, and establishing nationwide protocols to ensure uniform implementation across healthcare institutions. Additionally, incorporating pharmacogenomics into medical education and professional training programs could foster a culture of personalized medicine. These measures would enhance early detection of DPD deficiency, minimize severe toxicity risks, and improve overall treatment outcomes.

Despite promising awareness levels, the lack of widespread implementation and uniformity in practices highlights the pressing need for actionable measures. Addressing these challenges through targeted training, policy reforms, and resource allocation could significantly enhance patient safety and treatment outcomes. Building on these insights, the conclusion will outline practical recommendations and propose avenues for future research to bridge these gaps effectively.

To ensure patient safety and align with international best practices, the Palestinian Ministry of Health should take concrete steps to promote the integration of DPD testing into clinical practice. Establishing national guidelines mandating DPYD genotyping or DPD enzyme activity testing for patients receiving fluoropyrimidines is essential, with a focus on high‐risk groups such as those with a history of severe toxicity, renal or hepatic impairment, or a family history of DPD deficiency. Oncology protocols should incorporate mandatory pretreatment DPD testing for colorectal, gastric, and breast cancer patients receiving 5‐FU or capecitabine. Additionally, investments should be made to expand laboratory infrastructure, ensuring that molecular and biochemical diagnostic facilities are equipped to conduct DPYD testing, alongside training programs for HCPs to properly interpret and utilize results. To increase awareness and adherence, educational campaigns and continuing medical education (CME) programs should be implemented, highlighting the role of DPD testing in preventing severe adverse drug reactions. Given financial constraints, the Palestinian Ministry of Health should explore subsidies, public–private partnerships, and integration into health insurance schemes to make testing more accessible and affordable. Finally, a national registry should be established to track testing uptake, FP toxicity cases, and clinical outcomes, enabling continuous policy refinement and improved accessibility over time. Implementing these measures will enhance patient safety, optimize FP therapy, and reduce the incidence of severe drug‐related toxicity in Palestine.

## 5 Conclusions

This study highlights critical gaps in the understanding and implementation of pharmacogenomics among HCPs in Palestine, especially oncologists and clinical pharmacists, regarding the prescription and management of FPs. While there is a significant level of knowledge about DPD deficiency and its implications, this awareness does not consistently translate into practice. The absence of comprehensive DPD deficiency screening before prescribing FPs increases the risk of toxicity, leading to severe complications such as hospitalizations or life‐threatening adverse effects. Key barriers contributing to this issue include limited resources, insufficient protocols, and the absence of standardized guidelines.

To address these gaps, it is imperative that healthcare institutions, policymakers, and educational bodies take immediate steps to integrate DPD testing into routine oncology practice. This can be achieved through the development of standardized protocols, investment in accessible diagnostic resources, and the implementation of targeted educational programs to enhance HCPs' competency in pharmacogenomics. Additionally, future research should explore the cost‐effectiveness of DPD testing in Palestine and assess the impact of targeted educational interventions on increasing testing rates and improving patient safety.

Additionally, policymakers and healthcare leaders must prioritize the integration of pharmacogenomic testing into clinical practice to align with international standards and enhance patient outcomes. A concerted effort to establish clear guidelines, improve institutional infrastructure, and promote awareness is essential to advancing personalized medicine in Palestine. The time to act is now—by strengthening pharmacogenomic practices, we can significantly improve cancer treatment safety and efficacy, ensuring better care for patients.

While this study provides valuable insights into the gaps in pharmacogenomics implementation, it is limited by its sample size and geographic scope, warranting future research involving larger and more diverse populations to explore additional barriers to DPD testing. To address these challenges, collaboration with international organizations and the initiation of pilot training programs could serve as practical first steps to enhance knowledge and establish best practices. Furthermore, these findings emphasize the urgent need for national guidelines that standardize DPD testing prior to FP therapy, ensuring consistent clinical practice and reducing toxicity risks. Integrating such testing into existing healthcare protocols would not only optimize patient safety and treatment outcomes but also align Palestine's healthcare practices with international standards, advancing the implementation of personalized medicine in oncology.

While this study provides valuable insights into the gaps in pharmacogenomics implementation, it is subject to certain limitations. The sample size, though adequate, may not fully represent all oncology practitioners in Palestine, potentially limiting the generalizability of the findings. Additionally, the study's cross‐sectional design restricts the ability to establish causal relationships between barriers to DPD testing and clinical outcomes. Moreover, the geographic scope of the study may not capture variations in institutional practices across different healthcare settings. Future research involving larger and more diverse populations, as well as longitudinal studies, would help further explore additional barriers and facilitators of DPD testing adoption. To address these challenges, collaboration with international organizations and the initiation of pilot training programs could serve as practical first steps to enhance knowledge and establish best practices. Furthermore, these findings emphasize the urgent need for national guidelines to standardize DPD testing prior to FP therapy, ensuring consistent clinical practice and reducing toxicity risks. Integrating such testing into existing healthcare protocols would not only optimize patient safety and treatment outcomes but also align Palestine's healthcare practices with international standards, advancing the implementation of personalized medicine in oncology.

## Author Contributions

**Asmahan Alzeer:** formal analysis (equal), investigation (equal), writing – original draft (equal).

## Ethics Statement

The study was conducted in accordance with the Declaration of Helsinki and approved by the Ethics of Al‐Quds University (REF 13/24, Date: 17/2/2024). for studies involving humans.

## Consent

Informed consent was obtained from all subjects involved in the study.

## Conflicts of Interest

The authors declare no conflicts of interest.

## Data Availability

Data is available upon request from the corresponding author.

## References

[1] S. N. Hoon , P. K. H. Lau , A. M. White , M. K. Bulsara , P. D. Banks , and A. D. Redfern , “Capecitabine for Hormone Receptor‐Positive Versus Hormone Receptor‐Negative Breast Cancer,” Cochrane Database of Systematic Reviews 2021 (2021): 10–11. 10.1002/14651858.CD011220.pub2 PMC8150746 34037241

[2] J. W. G. Derksen , K. C. Smit , A. M. May , and C. J. A. Punt , “Systematic Review and Non‐Inferiority Meta‐Analysis of Randomised Phase II/III Trials on S‐1‐Based Therapy Versus 5‐Fluorouracil‐ or Capecitabine‐Based Therapy in the Treatment of Patients With Metastatic Colorectal Cancer,” European Journal of Cancer 166 (2022): 73–86. 35279472 10.1016/j.ejca.2022.02.004

[3] S. Glewis , M. Alexander , M. N. H. Khabib , et al., “A Systematic Review and Meta‐Analysis of Toxicity and Treatment Outcomes With Pharmacogenetic‐Guided Dosing Compared to Standard of Care BSA‐Based Fluoropyrimidine Dosing,” British Journal of Cancer 127 (2022): 126–136, 10.1038/s41416-022-01779-6. 35306539 PMC9276780

[4] Y. Cura , C. Pérez‐Ramírez , A. Sánchez‐Martín , et al., “Influence of Single‐Nucleotide Polymorphisms on Clinical Outcomes of Capecitabine‐Based Chemotherapy in Colorectal Cancer Patients: A Systematic Review,” Cancers (Basel) 15 (2023): 5–21. 10.3390/cancers15061821 PMC10046456 36980706

[5] M. J. Deenen , D. Meulendijks , A. Cats , et al., “Upfront Genotyping of DPYD*2A to Individualize Fluoropyrimidine Therapy: A Safety and Cost Analysis,” Journal of Clinical Oncology 34 (2016): 227–234, 10.1200/JCO.2015.63.1325. 26573078

[6] U. Amstutz , S. Farese , S. Aebi , and C. R. Largíadér , “Dihydropyrimidine Dehydrogenase Gene Variation and Severe 5‐Fluorouracil Toxicity: A Haplotype Assessment,” Pharmacogenomics 10 (2009): 931–944, 10.2217/pgs.09.28. 19530960

[7] B. Yang , X. Xie , D. Lv , et al., “Capecitabine Induces Hand‐Foot Syndrome Through Elevated Thymidine Phosphorylase‐Mediated Locoregional Toxicity and GSDME‐Driven Pyroptosis That Can be Relieved by Tipiracil,” British Journal of Cancer 128 (2023): 219–231, 10.1038/s41416-022-02039-3. 36347964 PMC9902485

[8] D. Meulendijks , L. M. Henricks , G. S. Sonke , et al., “Clinical Relevance of DPYD Variants c.1679T>G, c.1236G>A/HapB3, and c.1601G>A as Predictors of Severe Fluoropyrimidine‐Associated Toxicity: A Systematic Review and Meta‐Analysis of Individual Patient Data,” Lancet Oncology 16 (2015): 1639–1650, 10.1016/S1470-2045(15)00286-7. 26603945

[9] B. Wörmann , C. Bokemeyer , T. Burmeister , et al., “Dihydropyrimidine Dehydrogenase Testing Prior to Treatment With 5‐Fluorouracil, Capecitabine, and Tegafur: A Consensus Paper,” Oncology Research and Treatment 43 (2020): 628–636, 10.1159/000510258. 33099551

[10] G. Gentile , A. Botticelli , L. Lionetto , et al., “Genotype‐Phenotype Correlations in 5‐Fluorouracil Metabolism: A Candidate DPYD Haplotype to Improve Toxicity Prediction,” Pharmacogenomics Journal 16 (2016): 320–325, 10.1038/tpj.2015.56. 26216193

[11] D. L. Hertz , D. M. Smith , S. A. Scott , J. N. Patel , and J. K. Hicks , “Response to the FDA Decision Regarding DPYD Testing Prior to Fluoropyrimidine Chemotherapy,” Clinical Pharmacology and Therapeutics 114 (2023): 768–779. 37350752 10.1002/cpt.2978

[12] C. Li , S. Ngorsuraches , C. Chou , L. Chen , and J. Qian , “Risk Factors of Fluoropyrimidine Induced Cardiotoxicity Among Cancer Patients: A Systematic Review and Meta‐Analysis,” Critical Reviews in Oncology/Hematology 162 (2021): 10–14. 10.1016/j.critrevonc.2021.103346 33930532

[13] G. Argilés , J. Tabernero , R. Labianca , et al., “Localised Colon Cancer: ESMO Clinical Practice Guidelines for Diagnosis, Treatment and Follow‐Up,” Annals of Oncology 31 (2020): 1291–1305, 10.1016/j.annonc.2020.06.022. 32702383

[14] A. Cervantes , R. Adam , S. Roselló , et al., “Metastatic Colorectal Cancer: ESMO Clinical Practice Guideline for Diagnosis, Treatment and Follow‐Up,” Annals of Oncology 34 (2023): 10–32, 10.1016/j.annonc.2022.10.003. 36307056

[15] F. C. A. de Moraes , A. B. de Almeida Barbosa , V. K. T. Sano , F. A. Kelly , and R. M. R. Burbano , “Pharmacogenetics of DPYD and Treatment‐Related Mortality on Fluoropyrimidine Chemotherapy for Cancer Patients: A Meta‐Analysis and Trial Sequential Analysis,” BMC Cancer 24 (2024): 1210, 10.1186/s12885-024-12981-5. 39350200 PMC11441158

[16] B. Tamraz and A. P. Venook , “DPYD Pharmacogenetics: To Opt‐In or to Opt‐Out,” JCO Oncology Practice 20 (2024): 1009–1011, 10.1200/OP.24.00255. 38743915

[17] R. Nogueiras‐Álvarez and I. Pérez Francisco , “Pharmacogenetics in Oncology: A Useful Tool for Individualizing Drug Therapy,” British Journal of Clinical Pharmacology 90 (2024): 2483–2508, 10.1111/bcp.16181. 39077855

[18] K. Surprenant , L. Murray , K. Merritt , M. Hopkins , and J. McIntyre , “Give Patients the Choice to Test for DPD Deficiency Before Fluoropyrimidine Chemotherapy,” JCO Oncology Practice 21 (2025): 264–265, 10.1200/OP.24.00443. 39626133

[19] K. Traynor , “DPYD Testing Makes Fluoropyrimidine Chemotherapy Safer,” American Journal of Health‐System Pharmacy 81 (2024): e197–e198, 10.1093/ajhp/zxae058. 38491984

[20] T. T. Ho , D. M. Smith , C. L. Aquilante , et al., “A Guide for Implementing DPYD Genotyping for Systemic Fluoropyrimidines Into Clinical Practice,” Clinical Pharmacology and Therapeutics 117 (2025): 1194–1208, 10.1002/cpt.3567. 39887719 PMC11993294

[21] H. H. Sukkarieh , T. AlSagoor , M. Alnuhait , et al., “Awareness and Attitudes of Oncology Specialists Toward Dihydropyrimidine Dehydrogenase Testing in Saudi Arabia,” Cancer Reports 6 (2023): 3–6, 10.1002/cnr2.1704. PMC9939998 36806724

[22] C. C. Okechukwu , X. Ma , N. Sah , C. Mani , K. Palle , and W. H. Gmeiner , “Enhanced Therapeutic Efficacy of the Nanoscale Fluoropyrimidine Polymer CF10 in a Rat Colorectal Cancer Liver Metastasis Model,” Cancers (Basel) 16 (2024): 1360, 10.3390/cancers16071360. 38611037 PMC11011147

[23] N. Sah , A. A. Shaik , G. Acharya , et al., “Receptor‐Based Strategies for Overcoming Resistance in Cancer Therapy,” Receptor 3 (2024): 425–443, 10.3390/receptors3040021.

[24] N. A. Almashagbah , A. A. Mahasneh , and K. G. Bodoor , “Pharmacogenetic Study of the Dihydropyridine Dehydrogenase Gene in Jordanian Patients With Colorectal Cancer,” Asian Pacific Journal of Cancer Prevention 23 (2022): 3061–3069, 10.31557/APJCP.2022.23.9.3061. 36172669 PMC9810321

[25] Y. Zhou and V. M. Lauschke , “Comprehensive Overview of the Pharmacogenetic Diversity in Ashkenazi Jews,” Journal of Medical Genetics 55 (2018): 617–627, 10.1136/jmedgenet-2018-105429. 29970487

[26] E. Goljan , M. Abouelhoda , M. M. ElKalioby , et al., “Identification of Pharmacogenetic Variants From Large Scale Next Generation Sequencing Data in the Saudi Population,” PLoS One 17 (2022): e0263137, 10.1371/journal.pone.0263137. 35089958 PMC8797234

[27] N. J. Ahmed , A. S. Alrawili , and F. Z. Alkhawaja , “Knowledge and Awareness on Personalized Medicine Amongst Health Care Specialists and University Students in Health Colleges in Saudi Arabia,” Journal of Pharmaceutical Research International 32, no. 16 (2020): 177–183, 10.9734/jpri/2020/v32i1630741.

[28] M. Algahtani , “Knowledge, Perception, and Application of Pharmacogenomics Among Hospital Pharmacists in Saudi Arabia,” Risk Management and Healthcare Policy 13 (2020): 1279–1291, 10.2147/RMHP.S267492. 32904476 PMC7455604

[29] X. Nie , T. Jia , X. Hu , et al., “Clinical Pharmacists' Knowledge of and Attitudes Toward Pharmacogenomic Testing in China,” Journal of Personalized Medicine 12 (2022): 1348, 10.3390/jpm12081348. 36013297 PMC9410027

[30] T. Obara , S. Abe , M. Satoh , S. R. Gutiérrez Ubeda , S. Yoshimachi , and T. Goto , “Awareness Regarding Clinical Application of Pharmacogenetics Among Japanese Pharmacists,” Pharmacogenomics and Personalized Medicine 8 (2015): 35–41, 10.2147/PGPM.S71813. 25691815 PMC4325628

[31] C. A. T. C. Lunenburg , C. H. van der Wouden , M. Nijenhuis , et al., “Dutch Pharmacogenetics Working Group (DPWG) Guideline for the Gene–Drug Interaction of DPYD and Fluoropyrimidines,” European Journal of Human Genetics 28 (2020): 508–517, 10.1038/s41431-019-0540-0. 31745289 PMC7080718

[32] K. Koo , A. Pasternak , L. Henry , V. Sahai , and D. Hertz , “Survey of US Medical Oncologists' Practices and Beliefs Regarding DPYD Testing Before Fluoropyrimidine Chemotherapy,” JCO Oncology Practice 18 (2022): 3–6. 10.1200/OP.21.00874 PMC9191302 35239419

[33] M. De With , A. Sadlon , E. Cecchin , et al., “Implementation of Dihydropyrimidine Dehydrogenase Deficiency Testing in Europe,” ESMO Open 8, no. 2 (2023): 101197, 10.1016/j.esmoop. 36989883 PMC10163157

